# Complete chloroplast genome sequence and phylogenetic analysis of *Camellia fraterna*

**DOI:** 10.1080/23802359.2020.1841576

**Published:** 2020-12-24

**Authors:** Bo Yu, Ying-bo Sun, Xiao-fei Liu, Li-li Huang, Ye-chun Xu, Chao-yi Zhao

**Affiliations:** Environmental Horticulture Institute, Guangdong Academy of Agricultural Sciences, Guangdong Key Lab of Ornamental Plant Germplasm Innovation and Utilization, Key Laboratory of Urban Agriculture in South China, Ministry of Agriculture, Guangzhou, China

**Keywords:** Chloroplast genome, *Camellia fraterna*, phylogenetic analysis

## Abstract

*Camellia fraterna* belongs to the genus *Camellia* in the family Theaceae. We sequenced and analyzed the complete chloroplast genome of *C. fraterna* by Illumina sequencing in this study. The full length of the complete chloroplast genome is 156,902 bp, containing a pair of inverted repeat regions of 26,030 bp (IRa and IRb) separated by a large single-copy (LSC) region of 86,583 bp and a small single-copy (SSC) region of 18,259 bp. The *C. fraterna* chloroplast genome encodes 135 genes, comprising 87 protein-coding genes, 37 tRNA genes, eight rRNA genes, and three pseudogenes. This study will be useful for further study on genetic diversity and molecular breeding.

*Camellia fraterna* was planted in the Environmental Horticulture Research Institute of the Guangdong Academy of Agricultural Sciences (N23°23′, E113°23′, Guangzhou, China) (no. EHRIGAASC002); evergreen shrubs or small trees. The flower is small, white, and single-petaled with a strong aroma. It can be used for garden or potted ornamental viewing. It is also a precious breeding parent material for cultivating aromatic camellia varieties.

The chloroplast genome DNA of *C. fraterna* was extracted from young leaves. Covaris M220 (Covaris, Woburn, MA) was used for breaking the DNA into 300 bp fragments, and we constructed shotgun sequencing libraries according to the TruSeq™ DNA Sample Prep Kit for Illumina. Whole genome sequencing was executed using the Illumina NovaSeq platform (Illumina, San Diego, CA) (Genepioneer Biotechnologies Co. Ltd., Nanjing, China). Pair-end Illumina raw reads were cleaned from adaptors and barcodes and then quality filtered using Trimmomatic (Bolger et al. 2014). Then, reads were mapped to the chloroplast genome of the reference species (GenBank accession number: NC_024663), using Bowtie2 v2.2.4 (Langmead and Salzberg 2012) to exclude reads of nuclear and mitochondrial origins. *De novo* assembly to reconstruct the chloroplast genomes using SPAdes 3.6.1 (Bankevich et al. 2012), and chloroplast contigs were concatenated into larger contigs using Sequencher 5.3.2 (Gene Codes Inc., Ann Arbor, MI). A ‘genome walking’ technique, using the Unix ‘grep’ function, was used to find reads that could fill any gaps between contigs that did not assemble in the initial set of analyses (Souza et al. 2019). Misassembled contigs were corrected by Jellyfish v.2.2.3 (Marcais and Kingsford 2011). Annotation of the chloroplast genomes was generated by CpGAVAS (Liu et al. 2012) and a circular representation was drawn using the online tool OGDRAW (Lohse et al. 2007). The complete chloroplast genome sequence has been submitted to GenBank with the accession number of MT663342.

The length of chloroplast genome sequence of *C. fraterna* is 156,902 bp, including two inverted repeat regions (IRa and IRb, each 26,030 bp) separated by an large single-copy (LSC) (86,583 bp) region and an small single-copy (SSC) (18,259 bp) region. The GC content of the overall chloroplast genome, IR regions, LSC, and SSC is 37.34, 43.02, 35.33, and 30.58%, respectively. The GC content of the two IR regions is higher than those of the SSC and LSC, which is similar with *Celosia cristata* (Liu et al. 2020), *Spathiphyllum* ‘Parrish’ (Liu et al. 2019), and *Spathiphyllum cannifolium* (Liu et al. 2019). The chloroplast genome contains 135 genes in total, including 87 protein-coding genes, 37 tRNAs, eight rRNAs, and three pseudogenes.

The whole genome was used for phylogenetic tree analysis. First, we use MAFF v7.427 (Kazutaka et al. 2005) – auto mode to align each sequence. The gaps in the alignment were removed using the program trimAl with ‘-nogaps’ v 1.4 (Capella-Gutierrez et al. 2009). Finally, MrBayes v3.2.7 (Fredrik et al. 2012) was used to construct the phylogenetic tree (Figure 1). We found that *C. fraterna* is closely related to *C. reticulata*.

**Figure 1.**
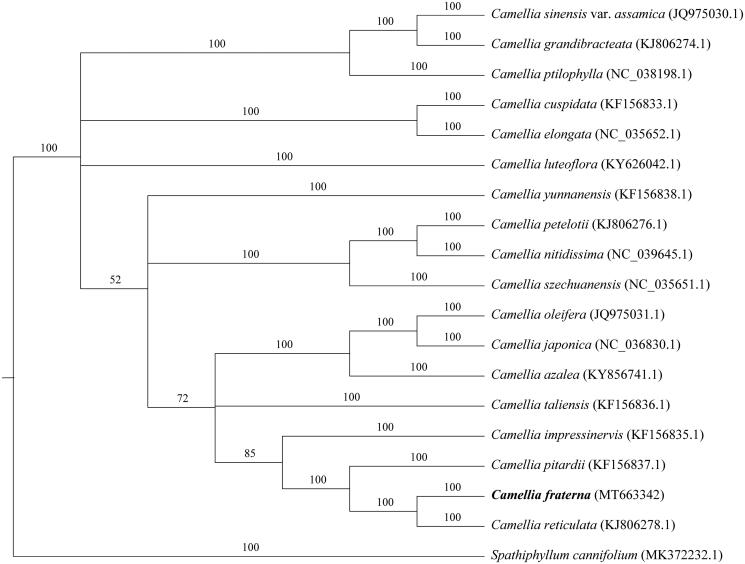
Phylogenetic tree reconstruction of 19 species based on sequences from whole chloroplast genomes. All the sequences were downloaded from NCBI GenBank.

## Data Availability

The data that newly obtained at this study are available in the NCBI under accession number of MT663342 (https://www.ncbi.nlm.nih.gov/nuccore/MT663342).
